# A Community-based Pulmonary Nodule Clinic: Improving Lung Cancer Stage at Diagnosis

**DOI:** 10.7759/cureus.4226

**Published:** 2019-03-11

**Authors:** Nathaniel Melton, John F Lazar, Troy A Moritz

**Affiliations:** 1 General Surgery, University of Pittsburgh Medical Center Pinnacle, Harrisburg, USA; 2 Cardiovascular Thoracic Surgery, University of Pittsburgh Medical Center Pinnacle, Harrisburg, USA

**Keywords:** pulmonary nodule clinic, lung cancer diagnosis, lung cancer screening, management of pulmonary nodules

## Abstract

Objective

Pulmonary nodules (PNs) are a common incidental finding and are often how lung cancer is discovered. Our goal was to determine if establishing a pulmonary nodule clinic (PNC) in a community healthcare setting would lead to an earlier stage at diagnosis.

Methods

A single healthcare system retrospective review was conducted of all PNC patients from 2010-2015 diagnosed with lung cancer. The stage at diagnosis was analyzed and compared to lung cancer patients in our healthcare system outside the PNC and to national data. Five-year survival rates for PNC patients from 2010-2012 were also analyzed.

Results

A total of 119 patients and 127 lung cancers were diagnosed through the PNC from 2010-2015. There were 990 lung cancers, with a known stage, diagnosed outside the PNC in our healthcare system from 2010 to 2015. Two hundred and eighty one (28.4%) cancers were Stage I, compared to 69 (54.3%) (p <0.0001) through the PNC; 110 (11.1%) cancers were diagnosed at Stage II compared to 17 (13.4%) through the PNC (0.4471); 277 (25.7%) cancers were diagnosed at Stage III, compared to 21 (16.5%) through the PNC (p 0.0060); 598 (60.4%) cancers were diagnosed at Stage IV, compared to 20 (15.7%) through the PNC (p <0.0001). Five-year survival rates for patients diagnosed in 2010 were 80% (four of five patients), 79.2% (19/24) in 2011, and 62.2% (23/37) in 2012.

Conclusions

Lung cancer survival is directly related to the stage at diagnosis. Establishment of our PNC has led to an earlier stage at diagnosis compared to the general lung cancer population in our community.

## Introduction

Cancer is the second leading cause of death in the United States with an overall survival of less than 20%, making it a significant public health concern. Patients often present with advanced disease, but improved survival rates are achieved with early detection and diagnosis at an early stage [1].

Effective screening methods like colonoscopy and mammogram have led to earlier detection and improved survival in colorectal and breast cancer respectively, prompting an exhaustive search for an equally useful screening tool for detecting lung cancer. The National Lung Screening Trial conducted by the American College of Radiology Imaging Network and the Lung Screening Study Group showed reduced mortality from lung cancer in high-risk patients with the use of computed tomography (CT) in comparison to chest radiography [2].

Pulmonary nodules (PN) are common incidental findings with a prevalence of 29% on routine CT. Additionally, the rate of detection of nodules is expected to improve with the advancement in CT technology and updated guidelines for lung cancer screening [2-3]. The nodules can vary in presentation from solitary pulmonary nodules (SPNs) to masses, to ground-glass opacities, and multiple nodules. The PN in the majority of the cases are benign, but the probability of these being malignant cannot be undermined [4-5]. Early identification of malignant nodules could lead to early diagnosis and increased survival. We sought to determine whether the establishment of a pulmonary nodule clinic (PNC) in a community hospital setting, led by a team of thoracic surgeons, could aid in early-stage diagnosis.

## Materials and methods

Our healthcare system consists of eight acute care hospitals and affiliated outpatient medical offices that provide care in Central Pennsylvania with our PNC catering to approximately 150 patients per year. The patient population is expected to increase in the future as insurers intend to cover lung cancer screenings. Our clinic included patients who had PN discovered while undergoing CT scans for lung cancer screening or as an incidental finding while undergoing diagnostic testing for unrelated medical conditions.

Institutional Review Board approval and patient consent waiver were obtained, and a single center retrospective review of patients from 2010-2015 diagnosed with lung cancer through our PNC was conducted. The medical and radiological data for each of these patients were retrieved from a running database of the PNC roster and associated cancer registry. The stage at diagnosis in non-PNC patients, during the same period, using chart review of our institutions' electronic medical record was also collected. The stage at diagnosis in our PNC patients was then compared to non-PNC patients and national data, as reported by the National Cancer Institute’s Surveillance, Epidemiology, and End Results Program (SEER), 7th edition. The five-year survival rates for our PNC patients from 2010-2012 were calculated using an electronic chart review.

Continuous variables were reported as mean and range, and categorical variables as number (percent). The chi-square test was used to analyze differences between groups in the early detection of lung cancer. All the analyses were done by SAS 9.4 (SAS Institute, Cary NC). A p-value of < 0.05 was considered significant.

## Results

A total of 119 patients were diagnosed with lung cancer through PNC from 2010-2015. Of these, eight patients had more than one primary lung cancer, so a total of 127 lung cancers were diagnosed during this period. This group consisted of 65 (54.6%) females and 54 (45.4%) males, with an average age of 68 years (42-90 years). Amongst those diagnosed with cancer, 50 (39.8%) were current smokers, 63 (49.6%) were former smokers, and 13 (10.2%) had never smoked (Table 1).

**Table 1:** Patient characteristics

n=119		
Age - mean (range)	68.22	42 - 90
Gender (female) - no.%	65	54.62%
Tobacco Use - no.%		
Current	50	42.02%
Previous	63	52.94%
Never	6	5.04%

The pathological staging of the cancers at the time of diagnosis was stage I-69 (54.3%); stage II-17 (13.4%); stage III-21 (16.5%); and stage IV-20 (16.5%) (Table 2).

**Table 2:** Tumor characteristics of pulmonary nodule clinic patients

	n=127
	Total (Percent)
STAGE	
1A	57 (44.88%)
1B	12 (9.45%)
2A	9 (7.09%)
2B	8 (6.30%)
3A	18 (14.17%)
3B	3 (2.36%)
4	20 (15.75%)
SITE	
Upper Lobe	74 (58.27%)
Middle Lobe	8 (6.30%)
Lower Lobe	39 (30.71%)
Lingula	1 (0.79%)
Pleura	2 (1.57%)
Hilum	3 (2.36%)
LATERALITY	
Left	55 (43.31%)
Right	72 (56.69%)

Histology revealed adenocarcinoma in 60 (47.2%) patients, squamous cell carcinoma in 36 (28.4%), poorly differentiated non-small cell lung cancer (NSCLC) in 8 (6.3%), and neuroendocrine in 13 (10.2%) patients. Other histologies including mesothelioma and B-cell lymphoma, comprised the rest of the histologic diagnoses. Tissue was obtained via percutaneous biopsy by interventional radiology in 77 (60.6%) patients, navigational bronchoscopy with endobronchial ultrasound in 15 (11.8%), and surgical biopsy in 5 (3.94%) patients. Overall 70 (55.1%) patients did not undergo surgical resection, while 57 (44.9%) did. Of the patients who underwent surgery, 16 (28.1%) of them underwent minimally invasive resection, 39 (68.4%) underwent open resection, and 2 (3.51%) underwent pneumonectomy.

**Figure 1:**
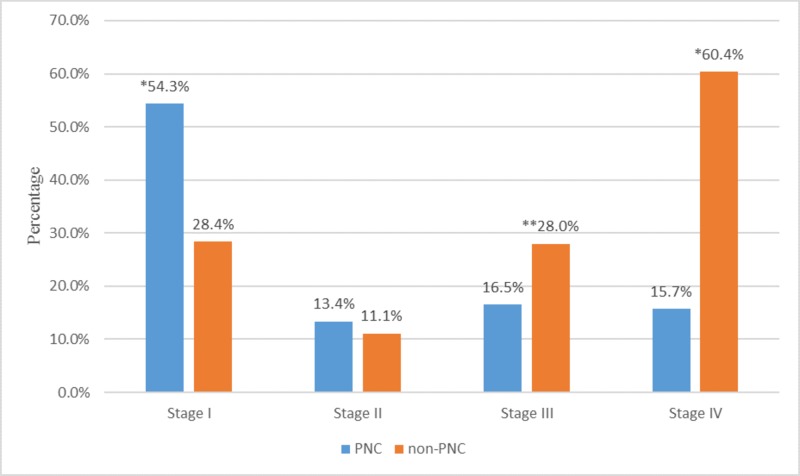
Comparisons of pathologic staging in pulmonary nodule clinic (PNC) and non-PNC patients (*p < 0.0001 **p < 0.05)

Through our healthcare system, 1,078 lung cancers were diagnosed from 2010 to 2015 outside the PNC. Of these, 88 patients had an unknown stage, with 990 having a known stage. Two hundred and eighty one (28.4%) patients were diagnosed at Stage I, compared to 69 (54.3%) (p < 0.001) through the PNC. One hundred and ten (11.1%) patients were diagnosed at Stage II, while 17 (13.4%) patients were diagnosed at Stage II through the PNC (p = 0.4471). Two hundred and seventy-seven patients were diagnosed at Stage III, compared to 21 (16.5%) through the PNC (p = 0.0060). Five hundred and ninety-eight (60.4%) patients were diagnosed at Stage IV, compared to 20 (15.7%) through the PNC (p < 0.0001) (Figure 1).

The five-year survival rate for PNC patients diagnosed in 2010 were 80% (four of five patients), 79.2% (19/24) in 2011, and 62.2% (23/37) in 2012. All patients diagnosed in 2010 and 2011 were stage I at diagnosis, while only 43% of patients diagnosed in 2012 were stage I at diagnosis.

## Discussion

In the United States, lung cancer occurs in approximately 225,000 patients and causes over 160,000 deaths annually. Even with advances in surgical techniques and medical therapies, the five-year survival rate remains around 20%. The survival rate is attributed to advanced disease at the time of presentation as in a majority of the cases lung cancer remains asymptomatic until it has metastasized. National data shows that 57% of lung cancer patients are stage IV at diagnosis [1].

Detection at an early stage is known to reduce malignancy-related mortality. Mammography and colonoscopy have led to a diagnosis at an earlier stage leading to improved survival rates for breast and colon cancer, respectively. It is expected that as is seen with other malignancies if lung cancer is caught at an earlier stage, it will lead to reduced mortality and improved survival outcomes [1, 6]. For decades, researchers have looked for a screening modality that could reduce lung cancer-related mortality. The National Lung Screening Trial showed that screening with low dose computed tomography could reduce lung cancer mortality in a select group of high-risk patients [2].

PNs are commonly encountered clinically and have both benign and malignant etiologies. The prevalence of PNs is unknown, and the rate of detection is expected to increase given the current lung cancer screening guidelines and increased use of CT scans [2-3]. The detection of PN is followed by the urgency to characterize it as benign or malignant. The probability of PN being malignant increases with age, pack-years of tobacco use, size of the nodule, and associated lymphadenopathy [7-8]. The American College of Chest Physicians has published guidelines for the management of PN based on the pretest probability of malignancy [4]. There are also extensive guidelines in the radiology literature regarding the management of PN which take into account the nodule size, morphology, growth rate, as well as patient-specific lung cancer risk factors [7, 9].

PNCs bring together thoracic surgeons, pulmonologists, radiation, and medical oncologists, interventional radiologists, and pathologists who collaborate to manage PN. These clinics are mostly found in academic, tertiary care centers. Our community PNC PN was founded in 2010 and is unique in that it was established and is driven by thoracic surgery. The goals of our clinic are to thoroughly evaluate each nodule, develop a comprehensive, individualized plan of care utilizing the most advanced technologies, and significantly reduce the time from detection to treatment. There is some recent data to suggest that the establishment of a dedicated PNC does indeed expedite the detection and treatment of lung cancer [10]. Through the establishment of our thoracic surgery led nodule clinic, we ultimately sought to detect lung cancer at an early stage and therefore improve the survival in our community.

**Figure 2:**
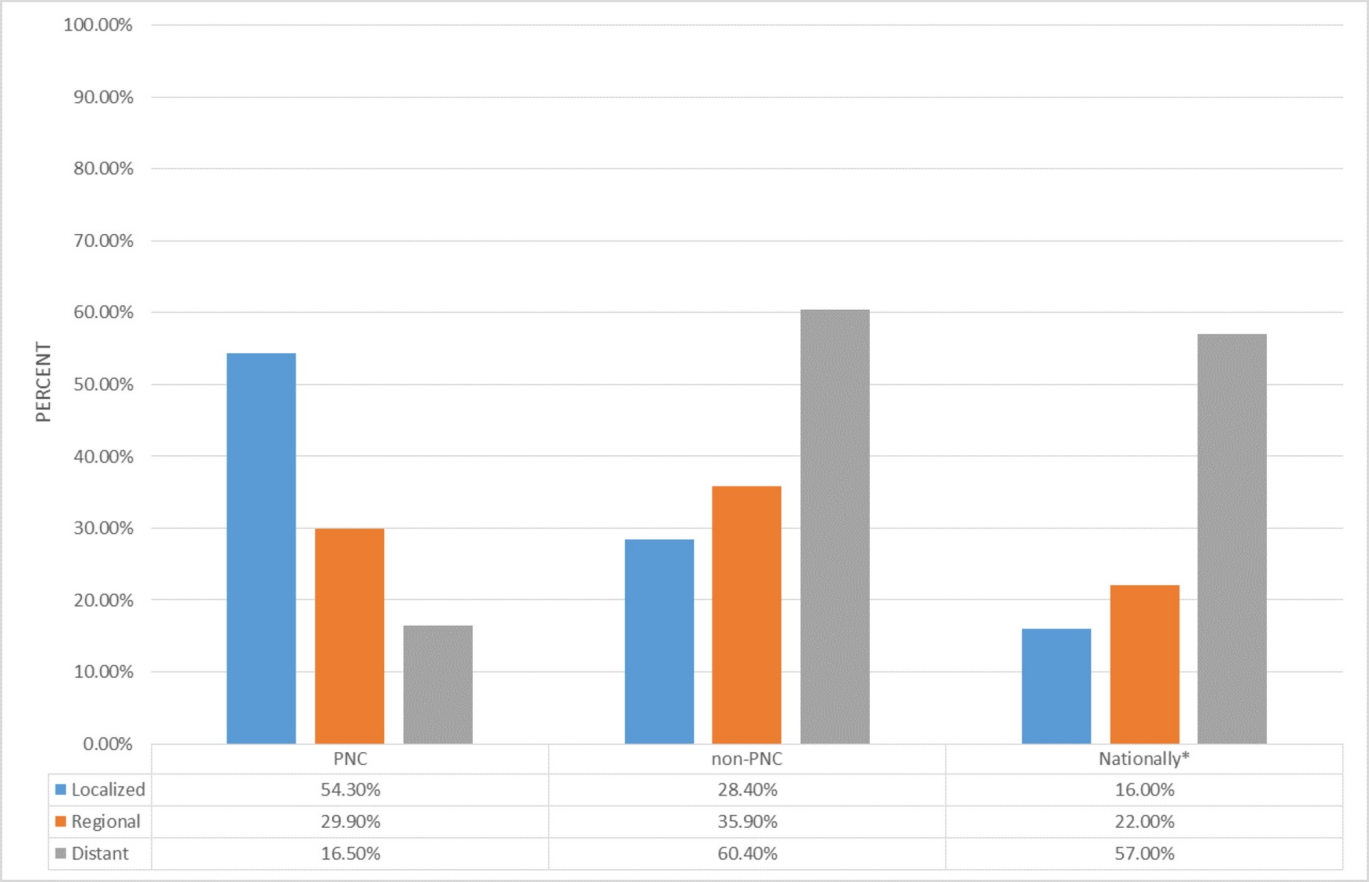
Stage at diagnosis for pulmonary nodule clinic (PNC) patients, non-PNC patients, and nationally (*As per the National Cancer Institute's Surveillance, Epidemiology, and End Results Program, 7th edition

From 2010-2015, we diagnosed 127 lung cancers in 119 patients that were referred to our clinic. Of these, 86 (67.7%) cancers were localized or regional at diagnosis in comparison to 391 (39.5%) cancers that were localized or regional at diagnosis in non-PNC patients. The most recent data from SEER shows that 38% of lung cancers are localized or regional at time of diagnosis nationwide [1] We believe these data show that the establishment of our PNC has positively benefited our community by leading to an early-stage diagnosis of lung cancer. The effect on mortality is yet to be determined and will be something that needs to be followed going forward (Figure 2). 

As mentioned previously, overall five-year survival remains around 20%. The five-year survival rates for clinic patients could be calculated for patients in 2010-2012. Of patients diagnosed in 2010, four out of five (80%) patients were diagnosed with Stage I lung cancer and were still alive at five years. Among the patients diagnosed in 2011, 19 out of 24 (79.2%) were alive at five years with 18 (94.7%) having at stage I disease at diagnosis. In 2012, 37 patients were diagnosed; 23 (62.2%) of these were still alive at five years with 16 (69.6%) having a stage I disease at diagnosis.

Interestingly, we found the average five-year survival rate of patients diagnosed at stage I, at our institution, during the five years before the establishment of our clinic to be 52%. This is in comparison to five-year survival rates in PNC patients diagnosed at stage I in 2010, 2011, and 2012, which were 100%, 94.7%, and 62.2%, respectively. While the reasons for these improved survival rates are most likely multifactorial, we feel that the establishment of our thoracic surgery run PNC has played a role in this. The PNC expedited the diagnosis, workup, and subsequent treatment of lung cancer in our community.

Going forward, given the lung cancer screening guidelines, an increasing number of people will be diagnosed with PNs. Consequently, PN evaluation has the potential to create a tremendous burden on individual patients and the health care system. It is essential that these patients be evaluated by clinicians experienced in dealing with and treating these nodules [11-12]. There are data that PNCs and lung cancer screening programs expedite the evaluation and treatment of lung cancer [10, 13]. In these clinics and screening programs, there is also data to support modifying risk factors to improve mortality [14].

Lastly, in a recent survey, most clinicians leading the evaluation of PNs were pulmonologists. Our PNC is unique, in that is driven by thoracic surgery. We believe that our study shows that thoracic surgery led PNC can be efficient and successful in evaluating and treating lung cancer. Furthermore, we believe that these data and our experience establishing our PNC, show that thoracic surgeons can and should lead the way in lung cancer screening and managing PNs.

Our study does have several limitations. First, we did not compare characteristics between patients diagnosed through the PNC and those diagnosed at the healthcare centers. So, it is possible that specific patient characteristics could account for significantly more patients having a stage IV diagnosis outside of the PNC. Also, the reason for the early-stage diagnosis through the PNC could be due to the lung cancer screening guidelines. We did not analyze if patients were referred to our clinic for lung cancer screening or further evaluation of PN found on imaging. There is also a significant selection bias, as the patients referred to the PNC were, by definition, referred there for PN evaluation. Lastly, the 7th edition of the American Joint Committee on Cancer lung cancer staging came out in around 2010, which could affect the Stage I survival rates before and after the establishment of our clinic.

## Conclusions

In conclusion, the PNC helped in diagnosing lung cancer at an earlier stage. This has been accomplished through the early detection of lung cancer, subsequent workup, and treatment. Not only did patients coming through the nodule clinic get diagnosed at an earlier stage compared to non-PNC patients, but they also were diagnosed at an earlier stage compared to the national data. A combination of PNC and the lung cancer screening guidelines can lead to earlier detections.

## References

[1] (2017). SEER Cancer Statistics Review, 1975-2014, National Cancer Institute. https://seer.cancer.gov/csr/1975_2014/..

[2] National Lung Screening Trial Research Team, Aberle DR, Adams AM (2011). Reduced lung-cancer mortality with low-dose computed tomographic screening. N Engl J Med.

[3] Gould MK, Tang T, Liu IL (2015). Recent trends in the identification of incidental pulmonary nodules. Am J Respir Crit Care Med.

[4] Tanner NT, Aggarwal J, Gould MK (2015). Management of pulmonary nodules by community pulmonologists: a multicenter observational study. Chest.

[5] Walter JE, Heuvelmans MA, de Jong PA (2016). Occurrence and lung cancer probability of new solid nodules at incidence screening with low-dose CT: analysis of data from the randomised, controlled NELSON trial. Lancet Oncol.

[6] Blandin Knight S, Crosbie PA, Balata H (2017). Progress and prospects of early detection in lung cancer. Open biol.

[7] Horeweg N, van Rosmalen J, Heuvelmans MA (2014). Lung cancer probability in patients with CT-detected pulmonary nodules: a prespecified analysis of data from the NELSON trial of low-dose CT screening. Lancet Oncol.

[8] Naidich DP, Bankier AA, MacMahon H (2013). Recommendations for the management of subsolid pulmonary nodules detected at CT: a statement from the Fleischner Society. Radiology.

[9] Holden VK, Wappel SR, Verceles AC (2017). Time to treatment outcomes from a dedicated pulmonary nodule clinic in B80-K thoracic oncology clinical outcomes. Am J Respir Crit Care Med.

[10] Wiener RS, Gould MK, Slatore CG (2014). Resource use and guideline concordance in evaluation of pulmonary nodules for cancer: too much and too little care. JAMA Intern Med.

[11] Golden SE, Wiener RS, Sullivan D (2015). Primary care providers and a system problem: a qualitative study of clinicians caring for patients with incidental pulmonary nodules. Chest.

[12] Miller DL, Mayfield WR, Luu TD (2016). Community-based multidisciplinary computed tomography screening program improves lung cancer survival. Ann Thorac Surg.

[13] Pastorino U, Boffi R, Marchiano A (2016). Stopping smoking reduces mortality in low-dose computed tomography screening participants. J Thorac Oncol.

[14] Simmons J, Gould MK, Laccarino J (2016). Systems-level resources for pulmonary nodule evaluation in the United States: a national survey. Am J Respir Crit Care Med.

